# *PIK3CA*-Related Overgrowth Spectrum From Diagnosis to Targeted Therapy: A Case of CLOVES Syndrome Treated With Alpelisib

**DOI:** 10.3389/fped.2021.732836

**Published:** 2021-09-09

**Authors:** Angelica Pagliazzi, Teresa Oranges, Giovanna Traficante, Chiara Trapani, Flavio Facchini, Alessandra Martin, Alessandro Semeraro, Anna Perrone, Cesare Filippeschi, Sabrina Giglio

**Affiliations:** ^1^Medical Genetics Unit, Meyer Children's University Hospital, Florence, Italy; ^2^Dermatology Unit, Department of Pediatrics, Meyer Children's University Hospital, Florence, Italy; ^3^Department of Pediatrics, Meyer Children's University Hospital, Florence, Italy; ^4^Department of Plastic Surgery, Meyer Children's University Hospital, Florence, Italy; ^5^Department of Radiology, Meyer Children's University Hospital, Florence, Italy; ^6^Department of Medical Sciences and Public Health, University of Cagliari, Cagliari, Italy

**Keywords:** *PIK3CA*, PROS, CLOVES, Alpelisib/BYL719, targeted (selective) treatment

## Abstract

*PIK3CA*-related overgrowth spectrum (PROS) is an umbrella term referring to various clinical entities, which share the same pathogenetic mechanism. These conditions are caused by somatic gain-of-function mutations in *PIK3CA*, which encodes the 110-kD catalytic α subunit of PI3K (p110α). These *PIK3CA* mutations occur as post-zygotic events and lead to a gain of function of PI3K, with consequent constitutional activation of the downstream cascades (e.g., AKT/mTOR pathway), involved in cellular proliferation, survival and growth, as well as in vascular development in the embryonic stage. *PIK3CA*-related cancers and PROS share almost the same *PIK3CA* mutational profile, with about 80% of mutations occurring at three hotspots, E542, E545, and H1047. These hotspot mutations show the most potent effect on enzymatic activation of PI3K and consequent downstream biological responses. If present at the germinal level, these gain-of-function mutations would be lethal to the embryo, therefore we only see them in the mosaic state. The common clinical denominator of PROS disorders is that they are sporadic conditions, presenting with congenital or early childhood onset overgrowth with a typical mosaic distribution. However, the severity of PROS is highly variable, ranging from localized and apparently isolate overgrowth to progressive and extensive lipomatous overgrowth associated with life-threatening vascular malformations, as seen in CLOVES syndrome. Traditional therapeutic approaches, such as sclerotherapy and surgical debulking, are often not curative in PROS patients, leading to a recrudescence of the overgrowth in the treated area. Specific attention has been recently paid to molecules that are used and studied in the oncogenic setting and that are targeted on specific alterations of the pathway PI3K/AKT/mTOR. In June 2018, Venot et al. showed the effect of Alpelisib (BYL719), a specific inhibitor for the p110α subunit of PI3K, in patients with PROS disorders who had severe or life-threatening complications and were not sensitive to any other treatment. In these cases, dramatic anatomical and functional improvements occurred in all patients across many types of affected organ. Molecular testing in PROS patients is a crucial step in providing the conclusive diagnosis and then the opportunity for tailored therapy. The somatic nature of this group of diseases makes challenging to reach a molecular diagnosis, requiring deep sequencing methods that have to be performed on DNA extracted from affected tissue. Moreover, even analyzing the DNA extracted from affected tissue there is no guarantee to succeed in detection of the casual somatic mutation, since the affected tissue itself is highly heterogeneous and biopsy approaches can be burdened by incorrect sampling or inadequate tissue sample. We present an 8-year-old girl with CLOVES syndrome, born with a large cystic lymphangioma involving the left hemithorax and flank, multiple lipomas, and hypertrophy of the left foot and leg. She developed severe scoliosis. Many therapeutic approaches have been attempted, including Sildenafil treatment, scleroembolization, laser therapy, and multiple debulking surgeries, but none of these were of benefit to our patient's clinical status. She then started treatment with Rapamycin from May 2019, without significant improvement in both vascular malformation and leg hypertrophy. A high-coverage Whole Exome Sequencing analysis performed on DNA extracted from a skin sample showed a mosaic gain-of-function variant in the *PIK3CA* gene (p.H1047R, 11% of variant allele frequency). Once molecular confirmation of our clinical suspicion was obtained, after a multidisciplinary evaluation, we decided to discontinue Sirolimus and start targeted therapy with Alpelisib (50 mg/day). We noticed a decrease in fibroadipose overgrowth at the dorsal level, an improvement in in posture and excellent tolerability. The treatment is still ongoing.

## Introduction

Somatic gain-of-function mutations in *PIK3CA*, determining a constitutive activation of the PI3K/AKT/mTOR pathway, are among the main driver mechanisms in cancer and are also responsible for benign overgrowth syndromes, collectively known as *PIK3CA*-related overgrowth spectrum (PROS) disorders (1). The *PIK3CA* gene encodes the 110-kD catalytic α subunit of PI3K, which converts phosphatidylinositol (3,4)-bisphosphate to phosphatidylinositol (3,4,5)-triphosphate, leading at last to phosphorylation and activation of AKT, by PKD1. The activity of AKT ultimately results in the activation of the kinase mechanistic target of rapamycin complex 1 (mTORC1). PI3K/AKT/mTOR pathway is involved in many biological processes, including cell metabolism, proliferation, survival and growth: a deregulation of PI3K/AKT/mTOR pathway functioning, in sense of overactivity, promotes oncogenesis and tissue overgrowth (2, 3). The acronym PROS includes many phenotypes, showing overlapping clinical features, but divergent in severity. Typically, PROS disorders share a congenital or early infantile onset, a segmental tissue overgrowth and a mosaic distribution (4, 5). These overgrowth conditions are sporadic, since gain-of-function mutations in *PIK3CA* occur necessarily as postzygotic events, that would otherwise be embryonic lethal if present at a germinal level. CLOVES (Congenital Lipomatous Overgrowth, Vascular malformations, Epidermal nevi, and Skeletal anomalies) syndrome is probably the most severe and complex phenotype within the PROS spectrum and is characterized by the presence of congenital lipomatous asymmetric and progressive overgrowth of the trunk with associated vascular malformations, epidermal nevi, skeletal and spinal anomalies (6–8). Increasing overgrowth, spinal deformities and extensive vascular malformations can lead to life-threatening complications in patients with CLOVES syndrome (1). Treatment options for PROS patients substantially consist in surgical debulking procedures, which are often non curative, leading to a recrudescence of the overgrowth in the treated area, and not free from risks (9). Sharing the same pathogenetic mechanism with cancer, opens the path to the use of repurposed cancer therapies in patients affected by PROS disorders (2, 10). In particular, inhibition of constitutively activated p110α subunit of PI3K using targeted molecules, as BYL719 (Alpelisib), has proven to be effective in the conservative treatment of PROS patients (11).

We present a case of an 8-years-old girl affected by CLOVES syndrome, presenting with a large thoracic lymphangioma, scoliosis, multiple dorsal lipomas, acral deformities and leg hemihypertrophy. Based upon the impressive results reported by of Venot et al. in a cohort of patients with PROS and a poor response to Sirolimus treatment in our patient, we opted for the administration of Alpelisib (11). Herein, we will report the data after about 1 year of treatment.

## Case Presentation

**Figure 1:**
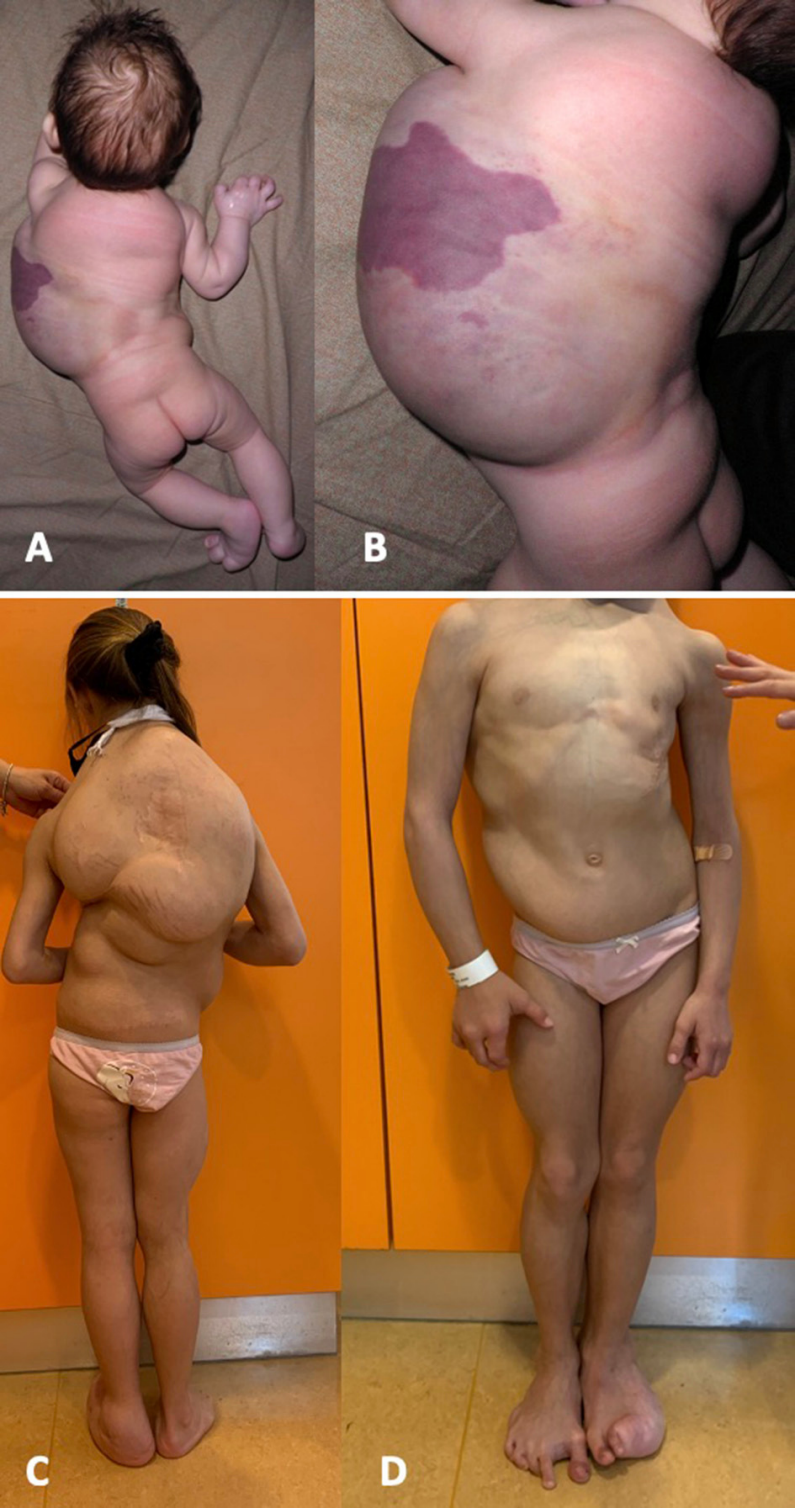
Representive pictures of our patient at birth **(A,B)** and at the age of 8 years **(C,D)**. She was born with a thoraco-abdominal large lymphangioma, covered by a capillary malformation, and hypertrophy of the left foot. She developed a severe dorsal scoliosis and a progressive overgrowth of the fibroadipose tissue, mainly localized at the trunk and at the left foot, with evident sandal gap at the right foot.

We present the case of an eight-years-old girl affected by CLOVES syndrome. She was born with cesarean section at 38 weeks of gestation with prenatal diagnosis of an extensive thoraco-abdominal lymphangioma. At birth, weight, length, occipitofrontal circumference were, respectively, 3,150 gr, 48 cm and 33 cm; Apgar scores at 1 min and 5 min were 9 and 10. Physical exam at birth revealed hypertrophy of the right hand and forearm, hypertrophy of the left foot with hypertrophy of the III, IV, and V digits on the same side, a sandal gap at the contralateral foot, and a large capillary malformation over the prenatally diagnosed thoracic lymphangioma (Figures 1A,B). She also had an interventricular defect, which was not hemodynamically significant. Clinical presentation at birth was misdiagnosed as Proteus syndrome. Magnetic resonance imaging (MRI) and ultrasounds of chest and abdomen confirmed the presence of a huge macrocystic lymphatic malformation, involving the dorsal paravertebral muscles and spinal canal and extended up to spleen and left kidney, and multiple dorsal lipomas. MRI study of the entire spinal column showed a dorsal scoliosis and lipoma of the filum terminale.

At 3 months of age our patient experienced the first of three scleroembolization procedures. She was treated with Sildenafil for 3 months, with no benefit.

After subsequent episodes of lymphatic malformation infection, at about 12 months of age, the giant lymphangioma was surgically removed. As with many complicated lymphatic malformations, radical surgery was not entirely feasible and our patient experienced reactivation and infection of the residual malformation. Meanwhile, to treat left foot deformities, III, IV, and V toes and IV metatarsal bones were disarticulated. Our patient was subjected to surgical debulking of the dorsal and left foot lipomas.

We performed a Whole Exome Sequencing (using SeqCap MedExome NimbleGen probes) on genomic DNA extracted from a tissue specimen, collected from the left foot overgrown area. Sequencing data showed the presence of *PIK3CA* (NM_006218) c.3140A>G (H1047R) gain-of-function mutation (11% mosaicism), confirming the diagnosis of CLOVES syndrome in our patient. The same variant was not identified in genomic DNA extracted from a blood sample. Given the persistence of residual thoraco-abdominal lymphatic malformation and regrowth of the removed lipomas, our patient started treatment with Sirolimus (0,8 mg/m^2^ twice a day), which in an allosteric inhibitor of mTORC1.

We did not notice a clear improvement, or a reduction of the overgrown tissues and our patient's clinical status remained substantially unvaried. After about 10 months of treatment, Sirolimus was suspended due to a Mycoplasma Pneumoniae infection.

Based upon Venot et al. research about the use of Alpelisib in patients with PROS, we decided to opt for this drug to treat the patient. Before starting the treatment, we performed cardiological evaluation, which was normal, MRI of chest, abdomen and left foot, and blood tests; the latter showed high levels of D-dimer, as typically seen in patients with CLOVES syndrome and vascular malformations, and slightly elevated bile acid levels, with normal lipase and gamma glutamyl transferase.

In April 2020, our patient started Alpelisib at the dosage of 50 mg/day. Subject to the favorable opinion of the ethics committee and signature of the informed consent by patient's parents, BYL719 was compassionately offered by Novartis following our request for nominal therapeutic use.

To monitor the response to treatment and occurrence of any adverse events, we repeated blood tests every 2 weeks for the first 3 months and then monthly and we performed MRI at third and sixth month of treatment. Spirometry showed measurements in the normal range, albeit at the lower limits, and did not highlight any restrictive deficits. For the first 2 weeks of treatment, a glucose sensor has been used to measure blood glucose concentration, which remained always in the normal range. Our patient is currently assuming Alpelisib without any adverse effects. Statural growth is processing regularly. We observed an improvement in posture, probably due to a reduction of the asymmetry between the lower limbs, and a moderate decrease of the volume of the dorsal lipomas and of the overgrown adipose tissue at the lumbar and left foot level. Moreover, basal D-dimer levels fell into the normal range (Supplementary Figure 1).

Physical measurements of the different segments, in order to monitor the response to treatment, were difficult to obtain in our patient given the complex and asymmetrical distribution of the fibroadipose overgrowth (Figures 1C,D): we noticed, with each physical examination, a change in shape and in the exact location of dorsal lipomas, which made it impossible to identify a fixed reference point for measurement.

**Figure 2:**
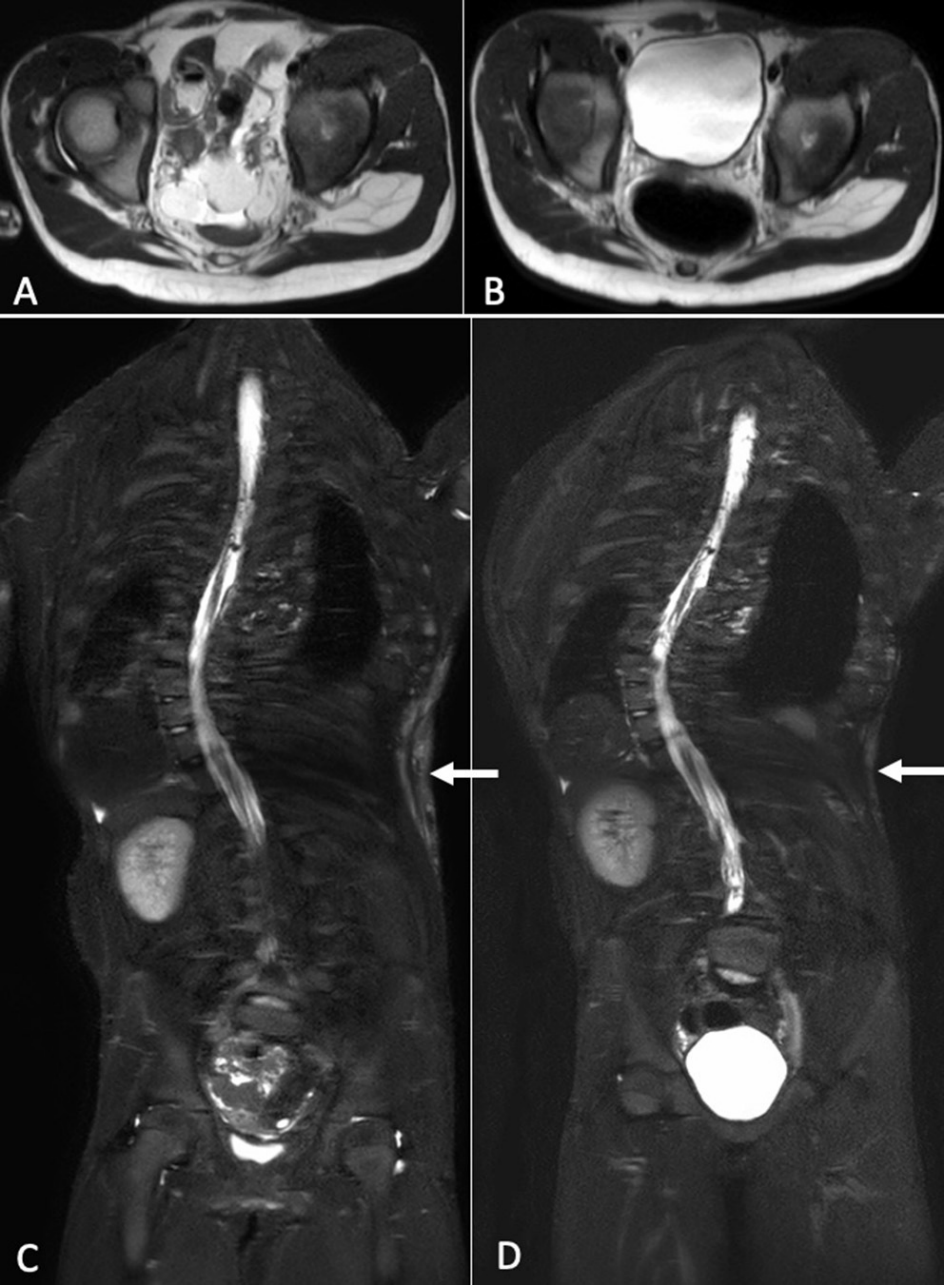
Axial T2-weighted MR images at baseline **(A)** and after about 6 months of therapy **(B)**. A reduction of the soft tissues and visceral lipomatous overgrowth can be observed after treatment. Coronal STIR-weighted MR images show high-signal-intensity alteration in the soft tissues of the left flank consisting with low-flow vascular malformation, at baseline **(C)** and after therapy **(D)**. After therapy, it was evident a reduction of the vascular malformation.

We compared MRI exam performed prior starting Alpelisib treatment with the same exam performed at about 6 months of therapy: we noticed a reduction of the volume of the fibroadipose tissue overgrowth in the dorsal area (Figures 2A,B) and a regression of the residual lymphatic malformation (Figures 2C,D).

In the course of Alpelisib treatment, a surgical debulking procedure to remove one of the dorsal lipomas was performed.

## Discussion

The term *PIK3CA*-related overgrowth spectrum embraces an increasing number of phenotypes, that we were used to consider as distinct clinical entities. A clear categorization of vascular malformations with or without segmental overgrowth based solely on the clinic is not always feasible, given the overlap of some characteristics between different disorders. It would be much easier to group vascular anomalies on the basis of a shared molecular pathogenesis: the term PROS, in fact, includes those conditions caused by the presence of a mosaic gain-of-function variant in the *PIK3CA* gene, which leads to an overactivity of the PI3K/AKT/mTOR pathway in the involved tissues. Therefore, achieving a molecular diagnosis is now even more relevant to choose the most appropriate therapy. Indeed, advance in the knowledge of the pathogenesis of segmental overgrowth syndromes and vascular malformations has also opened the path to consider a tailored pharmacological therapy for these conditions. The discovery that signaling pathways involved in oncogenesis and cancer progression are equally responsible of benign diseases, such as PROS disorders, has drawn attention to the possibility of use in these latter targeted molecules deriving from cancer scientific research. On the other hand, repurposing cancer therapies for treatment of vascular anomalies and segmental overgrowth syndrome has prompted scientists to revisit therapeutic approaches in cancer patients (12). Conventional therapeutic approaches, such as scleroembolization or surgical debulking procedures, are rarely curative in patients with vascular malformations and PROS disorders and are not devoid of risks of complications and recurrence. Since constitutive activation of the PI3K/AKT/mTOR pathway is a major cause of vascular anomalies and overgrowth syndromes, the use of mTOR inhibitors, such as Sirolimus, appeared as a good therapeutic strategy (4). Nevertheless, Sirolimus is a direct inhibitor of mTORC1, which is found at the end of the PI3K/AKT pathway: activation of mTORC1 is responsible for some but not all of the biological effects caused by PI3K gain-of-function. Indeed, PI3K, increasing the activity of AKT, determines the constitutional activation of other signaling cascades, which are not expected to respond to the inhibitory action of Sirolimus (Supplementary Figure 2). In a recent study, the authors measured outcomes and safety of a 26-week low-dose Sirolimus therapy in 39 patients with PROS disorders (13). Low-dose Sirolimus treatment exerted a greater effect on progressive overgrowth of the adipose tissue, rather than inducing a significant regression of existing overgrowth.

Sirolimus therapy was not free from adverse events and seven participants had to discontinue treatment: given the strong immunosuppressive activity of Sirolimus, the most common adverse events were infections, followed by lymphatic or blood disorders (13).

In 2018, Canaud's research group published the impressive results of treatment with Alpelisib in a group of 19 patients with CLOVES and other PROS conditions (11). Alpelisib is a molecule primarily developed for the treatment of *PIK3CA*-mutated cancers: it is a direct and specific inhibitor of the p110α subunit of PI3K, which is strongly and permanently activated due to the presence of a gain-of-function mutation in *PIK3CA*. Using a selective inhibitor of p110α, which is at the top of the PI3K/AKT/mTOR signaling pathway, it provides a direct outcome on affected tissues while reducing the risk of off-target effects. Either in children and in adult patients, Alpelisib was not associated with severe adverse events and patients are still under therapy, showing constant improvement of their condition (11, 14). In our patient's experience, after 12 months of treatment we observed an initial regression of the adipose overgrowth and of the low-flow vascular malformation, with a reduction of basal D-dimer levels and without adverse effects.

In conclusion, Alpelisib is a promising targeted treatment in patients with overgrowth syndromes, with or without vascular malformations, caused by an overactivity of PI3K.

According to the literature data available so far, Alpelisib has been shown to be a safe drug and, even after long periods of treatment, a progressive benefit has been noted, without resistance or tolerance to the action of the drug. Further studies in this field are needed to define the appropriate pro/kg dose of Alpelisib in order to obtain the best treatment result without the risk of adverse drug effects: for this purpose, a prospective phase II multi-center study is now recruiting pediatric and adult patients with PROS disorders to assess efficacy, safety and pharmacokinetics of Alpelisib (Clinical Trial NCT04589650).

Based on a shared molecular diagnosis, we could speculate that treatment with Alpelisib could be able to induce a reduction and an improvement also in isolated *PIK3CA*-related vascular malformations, such as venous or lymphatic malformations.

Considering the need for a definitive molecular diagnosis to access therapy and the detection rate of conventional sequencing methods in these somatic disorders, further studies are needed to implement molecular techniques in order to detect potential pathogenic *PIK3CA* variants.

## Data Availability Statement

The raw data supporting the conclusions of this article will be made available by the authors, upon reasonable request.

## Ethics Statement

This study was reviewed and approved by the Pediatric ethics committee of Meyer Children's University Hospital. Written informed consent to participate in this study was provided by patient's parents.

## Author Contributions

APa wrote the first draft of the manuscript, then all authors contributed to manuscript revision, read, and approved the submitted version. All authors have been involved in the multidisciplinary management of the patient.

## Conflict of Interest

The authors declare that the research was conducted in the absence of any commercial or financial relationships that could be construed as a potential conflict of interest.

## Publisher's Note

All claims expressed in this article are solely those of the authors and do not necessarily represent those of their affiliated organizations, or those of the publisher, the editors and the reviewers. Any product that may be evaluated in this article, or claim that may be made by its manufacturer, is not guaranteed or endorsed by the publisher.
