# Supplementary figures and images for: PIK3CA-Related Overgrowth Spectrum From Diagnosis to Targeted Therapy: A Case of CLOVES Syndrome Treated With Alpelisib
Source: Front Pediatr. 2021 Sep 9;9:732836. doi: 10.3389/fped.2021.732836 (PMC8459713; doi:10.3389/fped.2021.732836)

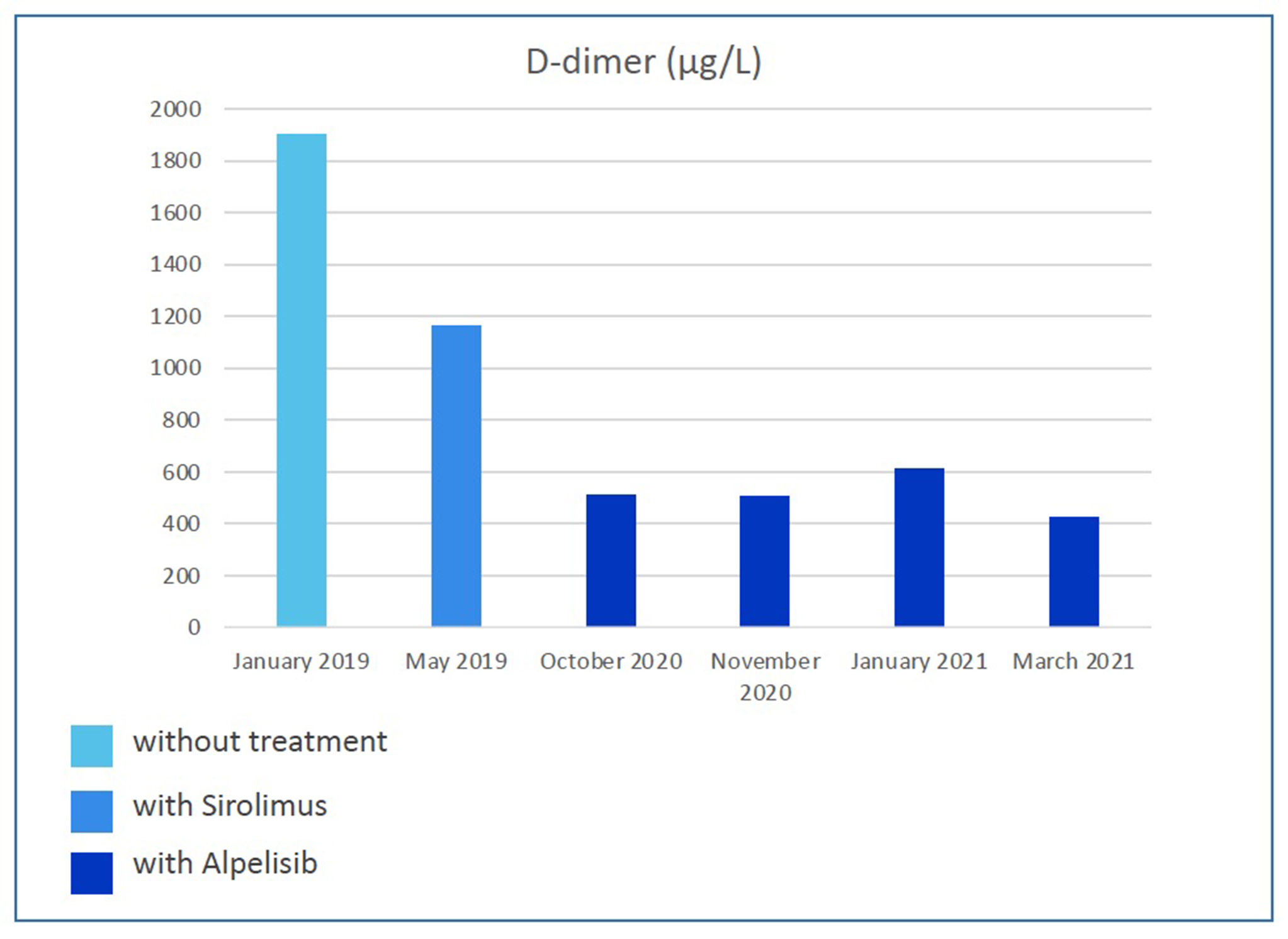

Supplement: Supplementary file 1 [file Image_1.JPEG]

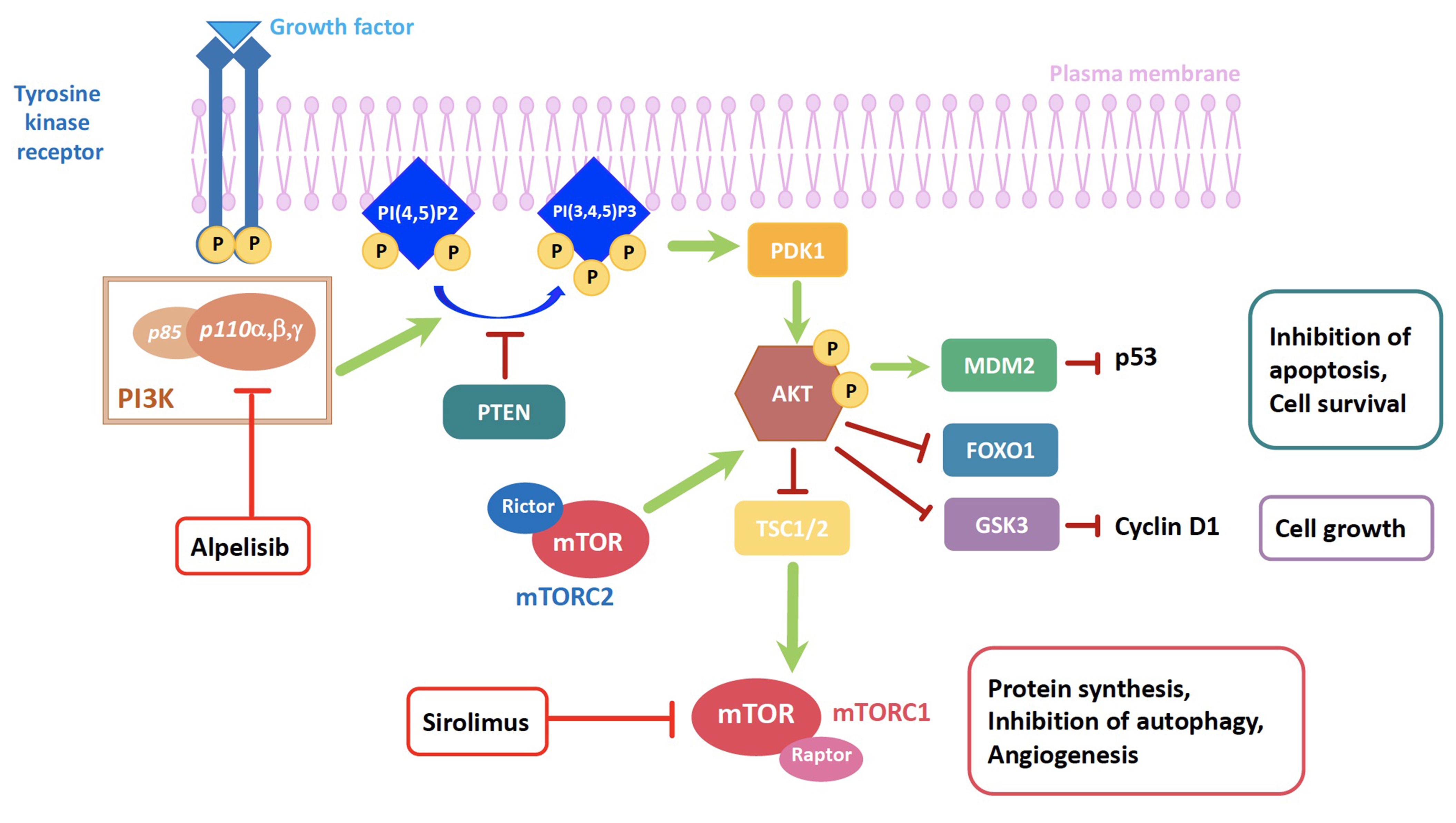

Supplement: Supplementary file 2 [file Image_2.JPEG]
